# Mutation screening in non-syndromic hearing loss patients with cochlear implantation by massive parallel sequencing in Taiwan

**DOI:** 10.1371/journal.pone.0211261

**Published:** 2019-01-25

**Authors:** Wei-Hsiu Liu, Pi-Yueh Chang, Shih-Cheng Chang, Jang-Jih Lu, Che-Ming Wu

**Affiliations:** 1 Department of Laboratory Medicine, Chang-Gung Memorial Hospital, Linkou Branch, College of Medicine, Chang-Gung University, Taoyuan, Taiwan; 2 Department of Otolaryngology—Head and Neck Surgery, Chang-Gung Memorial Hospital, Linkou Branch, College of Medicine, Chang-Gung University, Taoyuan, Taiwan; 3 Department of Medical Biotechnology and Laboratory Science, Chang-Gung University, Taoyuan, Taiwan; 4 Department of Medical Research, Chang-Gung Memorial Hospital and Graduate of Institute of Clinical Medical Science, Chang Gung University, Taoyuan, Taiwan; King Faisal Specialist Hospital and Research Center, SAUDI ARABIA

## Abstract

**Objectives:**

To explore the molecular epidemiology of rare deafness genes in Taiwanese sensorineural hearing impairment (SNHI) patients with cochlear implantation (CI) by performing massive parallel sequencing (MPS) and correlating genetic factors and CI outcomes.

**Methods:**

We enrolled 41 Taiwanese non-syndromic deafness patients with CI that lacked known mutations in common deafness genes. All probands were screened by a targeted exon amplification method that used massively parallel sequencing to screen a customized panel that included 40 relatively rare non-syndromic deafness genes.

**Results:**

Thirteen candidate variants in nine relatively rare deafness genes (*MYO15A*, *TMC1*, *MYH14*, *MYO3A*, *ACTG1*, *COL11A2*, *DSPP*, *GRHL2*, and *WFS1*) were identified in 24.4% (10/41) of the non-syndromic deafness probands with CI. According to the ACMG Standards and Guidelines, five variants in *MYO15A* and *ACTG1* were classified as likely pathogenic variants. Two of three multi-generational pedigrees exhibiting deafness were analyzed for the segregation of the disorder with the possible disease-causing variants. Patients with variants detected in most of the identified variant-bearing genes showed relatively good CI outcomes.

**Conclusions:**

We successfully identified candidate variants in partially deaf Taiwanese probands who lacked the known mutations in common deafness genes. Comparing the progress of hearing rehabilitation in CI patients with their apparent causative variants and the expression profiles of their altered genes allowed us to speculate on how alterations in specific gene sets may influence outcomes in hearing rehabilitation after CI.

## Introduction

Sensorineural hearing impairment (SNHI) is a common clinical disorder that severely to profoundly affects at least 1 in 1000 children of developed countries [1]. Cochlear implantation (CI) is currently regarded as the standard treatment for severe to profound SNHI in children. CI has well-documented benefits for spoken language, reading skills, and cognitive development [2], but the outcomes after CI can vary among individuals. Age at implantation [3, 4], residual hearing [5], the presence of inner ear malformations [6], the presence of cochlear nerve deficiency [7], parent-child interactions [2], and socioeconomic status [2] have all been shown to affect the outcomes.

Genetic factors contribute to SNHI in more than 50% of these patients [8]. To date, more than 100 genes and loci have been associated with deafness, and approximately 50 genes have been shown to cause non-syndromic hereditary hearing impairment (The Hereditary Hearing Loss Homepage, http://hereditaryhearingloss.org/) [9]. Due to limitations in mutation detection methodologies, most of the existing studies have focused on the three most common deafness genes, *GJB2* (or *Cx26*), *SLC26A4* (or *PDS*), and *MT-RNR1* [10], in the context of epidemiological studies or examining the correlation between CI outcomes and genotypes [11–14]. Only 1/3 of SNHI patients and 1/4 patients with CI can be identified as having known mutations in common deafness genes [10, 14]. The development of massively parallel sequencing (MPS), also known as next-generation sequencing, has allowed researchers and clinicians to more easily address such extraordinarily heterogenetic disorders [15–20]. The powerful strategy of simultaneously obtaining high-throughput reads from multiple targeted genes in numerous samples has reduced the sequencing cost and turnaround time of genetic examination. In recent years, MPS technology has been widely used to examine the prevalence of relatively rare deafness genes and the correlations between various genotypes and the outcomes of CI rehabilitation [15, 19–24].

The auditory performance in patients with CIs can be predicted according to the pathologies that are associated with mutations in different genes [14, 20–22, 24, 25]. When the function of the mutated gene is confined to the intra-cochlear etiology, good post-CI auditory performance is possible [14]. Conversely, poor CI performance can be predicted when the causative mutation occurs in a gene that is expressed in the spiral ganglion neurons (SGNs), brainstem auditory nuclei, or hair cell synapses. Genetic information about predicted good or poor CI outcomes could allow clinicians to counsel patients on whether to undergo an operation and/or decide on a rehabilitation program [24, 26]. Therefore, it is beneficial to identify the causative gene mutation prior to CI intervention. A greater knowledge of the genetic backgrounds of deafness patients with CI will enable clinicians to offer a more precise, genetically based prediction of CI outcome.

In this study, we sought to unveil the prevalence rate of rare deafness-associated variants in 41 Taiwanese SNHI patients with CIs, using MPS technology. We also examined the relationship between the identified variants and the outcomes of CI.

## Materials and methods

### Subject recruitment

A total of 41 probands who had bilateral hearing loss and lacked the known mutations in common deafness genes, including *GJB2*, *SLCA26A4* and m.1555A>G in *MT-RNR1* [27], were recruited from Chang Gung Memorial Hospital (CGMH). All participants had non-syndromic hearing loss without any other organic abnormality and had undergone unilateral CI at CGMH. The subjects included three probands from multiplex families and 38 simplex probands. Their average residual hearing before implantation was 103.3±11.4 dBHL (decibels Hearing Level). The subjects included 18 males (43.9%) and 23 females (56.1%). The average age at which they received CI was 6.6 years old (range, 0.9 years to 33.3 years).

The study protocol and written informed consent form were approved by the Chang-Gung Memorial Hospital Ethics Committee for Human Studies. Signed informed consent forms were obtained from all participants or their guardians before we began the testing procedures.

### Massively parallel sequencing

Genomic DNA (gDNA) was extracted from peripheral blood using a QIAamp DNA blood mini kit (Qiagen, Taiwan). DNA libraries were generated by the targeted exon amplification method. An Ion Ampliseq^TM^ Custom Panel of 40 relatively rare and non-syndromic deafness genes (14 autosomal dominant, 19 autosomal recessive, 6 autosomal dominant/recessive, and 1 X-linked; S1 Table) were selected for our customized panel (Applied Biosystems, Life Technologies, Carlsbad, CA) by Ion Ampliseq^TM^ Designer (Version 3.6, https://www.ampliseq.com/browse.action). This customized panel comprised 1319 amplicons with 5 bp exon padding, and covered 95.6% of the exonic regions. The whole panel size was 256.6 Kb. The 40 genes screened in this panel were selected from our survey, and the examined amplicons included all of the relevant gene variants identified in studies on populations from China, Korea, and Japan [17, 25, 28]. The gene set included *MYO15A*, *TMC1*, *PCDH15*, *CDH23*, *MYO7A*, *ESRRB*, *MARVELD2*, *TECTA*, *WHRN*, *MYO6*, *POU3F4*, *CDH23*, *COL11A2*, *WFS1*, *EYA4*, *STRC*, *TMPRSS3*, *WHRN*, *ACTG1*, *DFNA5*, and *CRYM*, in which mutations have been commonly found in hearing loss patients of Eastern Asia. For library preparation, we used an Ion Ampliseq Library Kit 2.0 (Applied Biosystems, Life Technologies) according to the manufacturer’s instructions. The purified amplicon libraries were assessed for their concentrations and size distributions using an Agilent Bioanalyzer 2100 (Agilent Technologies, Inc., Santa Clara, CA, USA). Each library was diluted to 10 pM and subjected to clonal amplification using an Ion OneTouch^TM^ 2 System and an Ion OneTouch 200 Template Kit v2 (Life Technologies). The obtained products were sequenced using an Ion Torrent Personal Genome Machine (PGM) system with an Ion PGM^TM^ 200 Sequencing Kit and an Ion 318^TM^ Chip or 316 ^TM^ Chip (Life Technologies). The average depth of coverage for the target region obtained for the 41 samples was 425.2-fold, and 96.2% of the targeted regions were read by at least 20× coverage.

### Data analysis

The raw sequence reads were processed using the Ion Torrent Suite^TM^ Software and aligned to a reference human genome sequence (Feb. 2009, GRCh37/hg19) with a Torrent Mapping Alignment Program optimized to the Ion Torrent^TM^ data. The variant calling process was conducted using the Torrent Variant Caller plug-in software (all from Life Technologies).

### Filtering criteria

**Fig 1:**
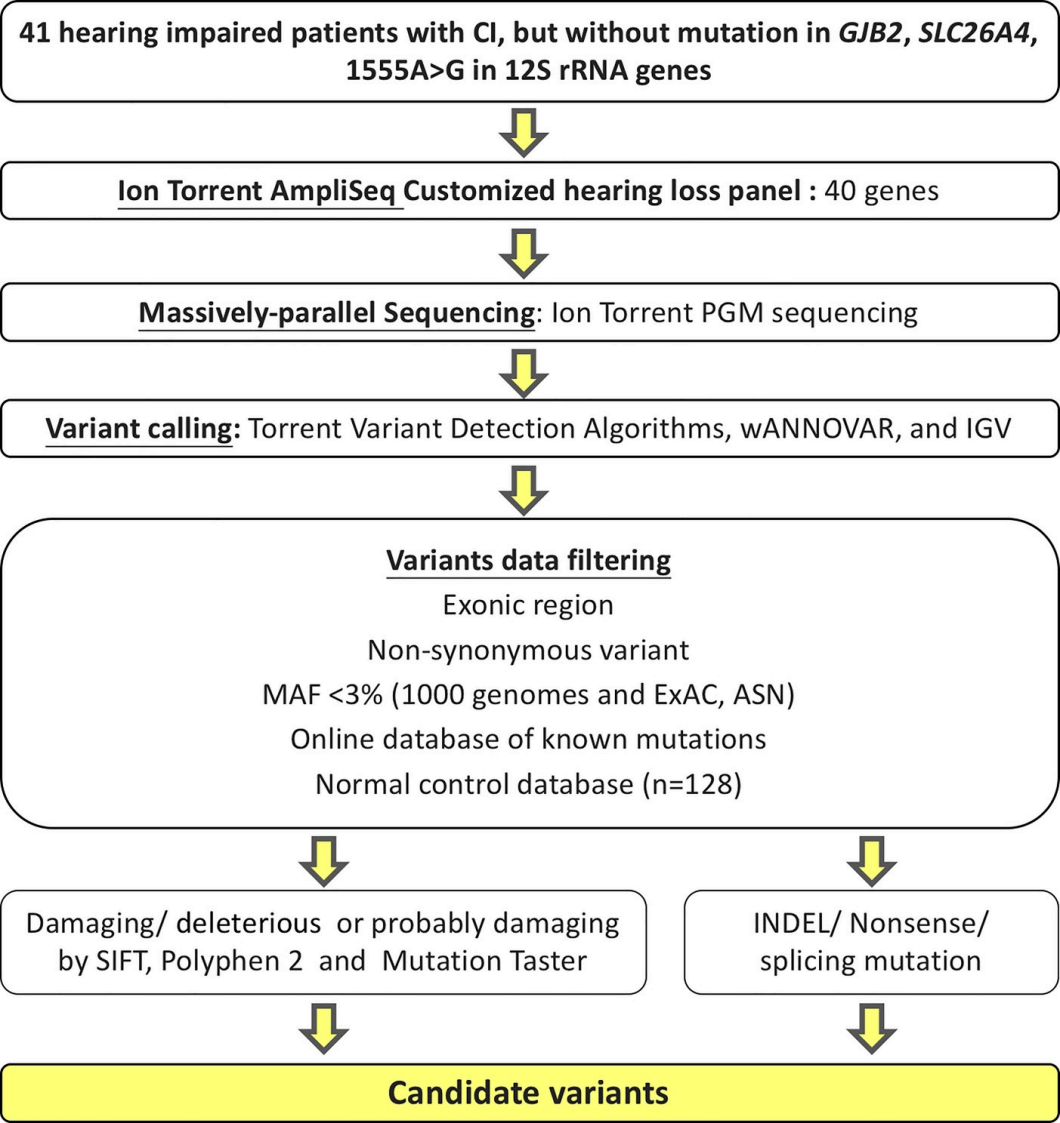
Pipeline used to identify candidate variants. Forty-one patients with CI who lacked the known mutations in common deafness-associated genes were subjected to comprehensive genetic analysis by using an Ion Torrent PGM sequencer to target 40 relatively rare deafness genes. Variants were called using plug-in Torrent variant detection algorithms, annotated through wANNOVAR, and initially confirm using the integrative Genome viewer. Annotated variants were filtered by various criteria, including: being located in an exonic region; being non-synonymous; having an allele frequency < 3% in the 1000 Genomes Project; having a variant frequency of 30–70% for heterozygotes or >85% for homozygotes; and being absent from online databases and 128 ethnically matched normal hearing controls. SIFT, Polyphen 2, and Mutation taster were used to predict the functions of the identified variants; we first filtered for missense variants, and then directly identified indels, splicing site variants, and nonsense variants.

Variants called by the Torrent Variant Caller were annotated through wANNOVAR, a web-based application for gene and amino acid annotation and functional evaluation [29, 30]. The following criteria were used to select variants: a non-synonymous variant in an exonic region; an allele frequency below 3% in the Asian population, as indicated by the 1000 Genomes Project [31] and Exome Aggregation Consortium (ExAC) [32], with a variant frequency of 30–70% for heterozygous variants and 85–100% for homozygous variants; allele coverage exceeding 30X; and variant allele coverage exceeding 15X. We also included variants that could lead to the synthesis of immature proteins (e.g., nonsense, INDEL, and splicing site variants) and missense variants that were nonsynonymous and annotated as “deleterious”, “damaging”, or “possibly damaging” by SIFT [33], Polyphen 2 [34], or Mutation taster [35] (Fig 1). All of the selected variants were further screened in 128 ethnically matched normal hearing controls (see Acknowledgements). Finally, bi-allelic variants selected for autosomal recessive genes were retained. The filtered variants were confirmed by Sanger sequencing and then taken as candidate variants.

### Evaluation of auditory and speech performance

To evaluate auditory performance in CI patients, we used the Categories of Auditory Performance (CAP) scale [36] and the Speech Intelligibility Rating (SIR) scale [37]. The CAP, which evaluates the auditory receptive ability, places an individual into one of eight categories ranging from “no awareness of environmental sound” (CAP score = 0) to “uses the telephone” (CAP score = 7). The SIR, which evaluates spontaneous speech intelligibility, places an individual into one of five categories ranging from “unintelligible speech” (SIR score = 1) to “speech intelligible to all listeners” (SIR score = 5). The CAP and SIR scales have been confirmed as reliable instruments for measuring CI outcomes [36, 37]. We referred to the literature to identify appropriate criteria [24], and classified a poor CI outcome as corresponding to CAP ≤ 5 and SIR ≤ 3.

## Result

### Identification of candidate variants

**Fig 2:**
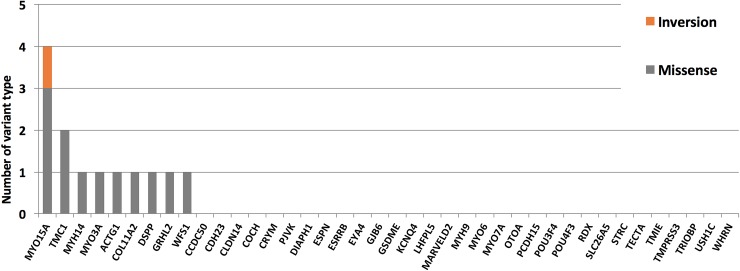
Candidate variants detected in nine genes of 41 patients. Each color bar indicates a different variant type, as indicated.

We identified a total of 13 previously unidentified candidate variants in nine deafness genes, as found in 10 of the 41 CI patients (24.4%) (Fig 1 and Table 1). The allele frequency of each of the 13 candidate variants was very low in the east Asian populations captured in the 1000 Genomes Project and ExAC, and these variants were all absent from our 128 normal Taiwanese controls. The 13 candidate variants included 12 missense variants in nine cases that were predicted to have damaging or disease-causing effects by causing amino acid changes, as assessed by protein-impact predictors, and one inversion variant found in one case (Table 1 and Fig 2). We identified four heterozygous variants responsible for autosomal dominant SNHI (ADSNHI) in four probands (DE3864, DE3335, DE3395, and DE4467). One homozygous variant and two compound heterozygous variants were consistent with recessive inheritance in two probands (DE3241 and DE4372). However, DE3386 harbored three candidate variants in genes whose mutations had previously been associated with dominant or recessive inheritance, making it impossible for us to conclusively determine how any one of the variants impacted the patient’s phenotype. Three probands (DE3221, DE4377, and DE4702) were identified as having three heterozygous variants in two genes, *COL11A2* and *TMC1*; as mutations in these genes had previously been associated with autosomal dominant and autosomal recessive inheritance patterns [38], we were unable to confirm the pathogenicity of either variant. The gene most frequently associated with a putatively pathogenic variant was *MYO15A* (4 variants), followed by *TMC1* (2), with the remaining genes each having a single putatively pathogenic variant (*MYH14*, *MYO3A*, *ACTG1*, *COL11A2*, *DSPP*, *GRHL2*, and *WFS1* (Fig 2). Beyond the variants specified above, five of the identified variants could be classified as likely pathogenic variants, and the remaining variants were of uncertain significance according to the AMA and ACMG guidelines [39].

**Table 1:** Thirteen different variants were identified in 10 patients.

Case	Gene	Variant type	Inheritance	Nucleotide change	Protein change	Genotype	SIFT	Polyphen2	Mutation Taster	1000 Genomes ASN	ExAC ASN	Normal control (allele fre, %)	Pathogenicity (Deafness variation database^a^)	AMA and ACMG guidelines
**DE2864**	*WFS1*	Missense	AD	NM_006005.3:c.173C>T	NP_005996.2:p.(Ala58Val)	Het	T	PD	N	0	0.0046	0	Pathogenic (Wolfram syndrome)	Uncertain significance
**DE3221**	*COL11A2*	Missense	AR/AD	NM_080680.2:c.191G>A	NP_542411.2:p.(Arg64Gln)	Het	T	PD	D	0.001	0.0011	0	VUS	Uncertain significance
**DE3241**	*MYO3A*	Missense	AR	NM_017433.4:c.1256T>C	NP_059129.3:p.(Ile419Thr)	Hom	D	B	D	0	0.0023	0	VUS	Uncertain significance
**DE3335**	*DSPP*	Inversion	AD	NM_014208.3:c.3021_3022inv	NP_055023.2:p.(Asp1008Asn)	Het	T	PD	P	0	0	0	-	Uncertain significance
**DE3386**	*MYH14*	Missense	AD	NM_024729.3:c.2080C>T	NP_079005.3:p.(Arg694Cys)	Het	D	D	D	0	0	0	-	Likely pathogenic
	*MYO15A*	Missense	AR	NM_016239.3:c.4457G>T	NP_057323.3:p.(Gly1486Val)	Het	D	D	D	0	0	0	-	Likely pathogenic
	*MYO15A*	Missense	AR	NM_016239.3:c.4101C>A	NP_057323.3:p.(Asn1367Lys)	Het	D	D	D	0	0	0	-	Likely pathogenic
**DE3395**	*ACTG1*	Missense	AD	NM_001199954.2:c.830C>T	NP_001186883.1:(p.Thr277Ile)	Het	D	D	D	0	0	0	-	Likely pathogenic
**DE4372**	*MYO15A*	Missense	AR	NM_016239.3:c.5443C>A	NP_057323.3:p.(Gln1815Lys)	Het	D	D	D	0	0	0	-	Uncertain significance
	*MYO15A*	Missense	AR	NM_016239.3:c.5977C>T	NP_057323.3:p.(Arg1993Trp)	Het	D	D	D	0	0	0	VUS	Likely pathogenic
**DE4377**	*TMC1*	Missense	AR/AD	NM_138691.2:c.1632T>G	NP_619636.2:p.(Phe544Leu)	Het	T	PD	D	0	0	0	VUS	Uncertain significance
**DE4467**	*GRHL2*	Missense	AD	NM_024915.3:c.193G>A	NP_079191.2:p.(Gly65Ser)	Het	T	PD	D	0	0	0	-	Uncertain significance
**DE4702**	*TMC1*	Missense	AR/AD	NM_138691.2:c.1777T>C	NP_619636.2:p.(Phe593Leu)	Het	D	B	D	0	0	0	-	Uncertain significance

^a^ Deafness variation database (http://deafnessvariationdatabase.org/)

B: Benign; T: Tolerated; D: Damaging/deleterious; PD: Probably damaging; NA: Not available; N: Polymorphism

VUS: Variant of uncertain significance

AD: Autosomal dominant; AR: Autosomal recessive

### Co-segregation analysis for multiplex probands

**Fig 3:**
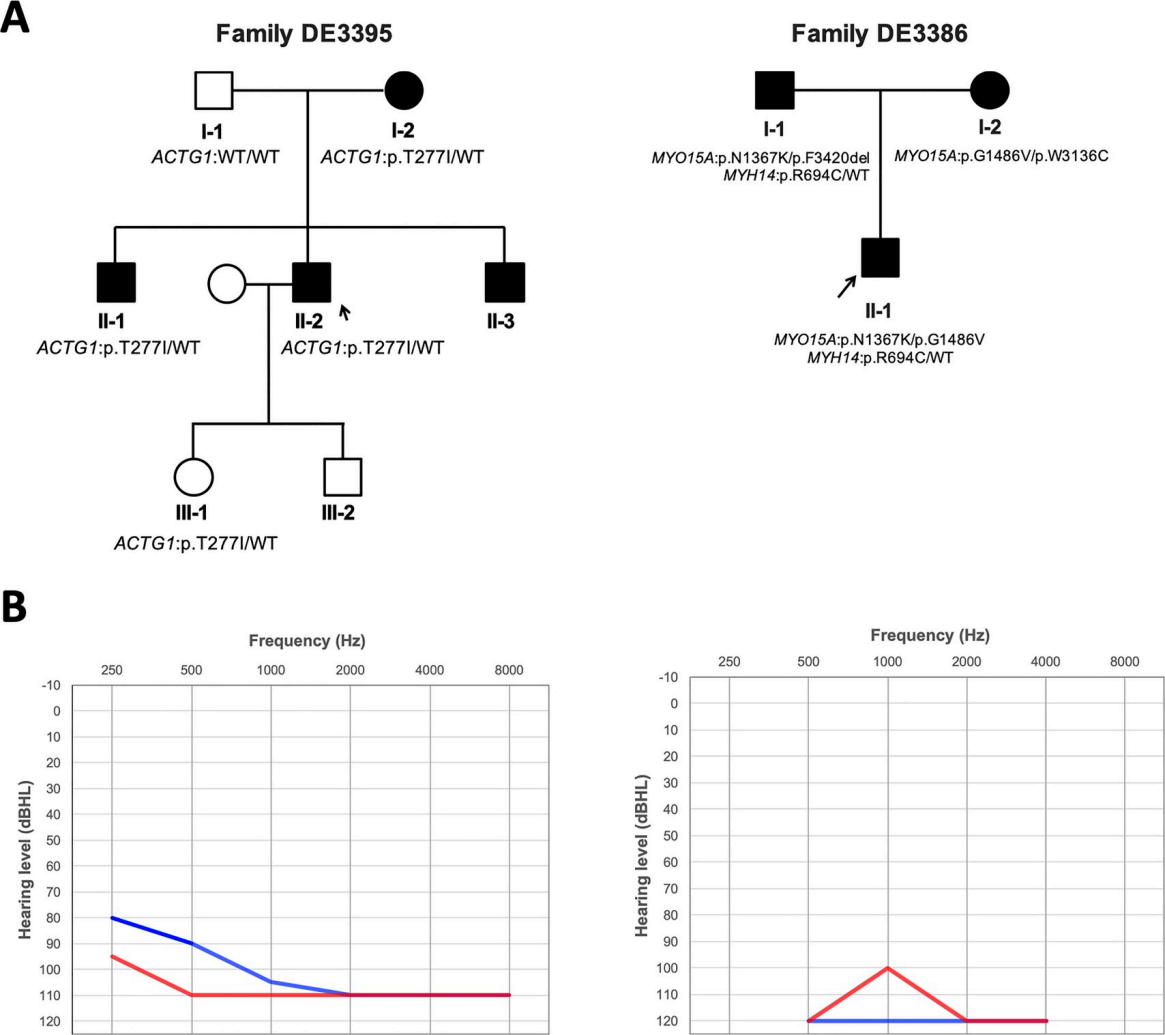
Families DE3395 and DE3386. (A) Probands of each family are indicated by arrows. (B) Audiograms of DE3395 (left) and DE3386 (right). Both recipients had bilateral symmetric flat-type audiograms of profound severity. Hearing levels of the right ear and left ear are marked with red and blue lines, respectively.

We identified four possible candidate variants in two of three multiplex probands, and performed segregation analysis using limited affected or unaffected familial members in Family DE3386 and Family DE3395 (Fig 3A and Fig 3B).

DE3395 is a late-onset hearing loss patient who was found to have p.(Thr277Ile) (c.830C>T) in *ACTG1* (Table 1). Among the family members of DE3395, his mother and two brothers also experienced a progressive deafness phenotype beginning in the first or second decade of life (as seen in the proband). Our direct sequencing identified the p.(Thr277Ile) variant in *ACTG1* in two additional affected members of this family (I-2 and II-1) and the proband’s daughter (III-1), who had a normal hearing ability at the age of 8 years. The final affected individual (II-3) did not consent to testing, so the genetic result was unavailable.

Three candidate variants, compound heterozygous variants (p.(Gly1486Val) and p.(Asn1367Lys)) in *MYO15A* and heterozygous variant (p.Arg694Cys) in *MYH14*, were identified in individual DE3386 (Table 1). Sanger sequencing revealed that p.(Arg694Cys) and p.(Asn1367Lys) were co-inherited from his father (I-1) and p.(Gly1486Val) came from his mother (I-2). Because both of his parents are also SNHI patients, we further applied MPS to reveal his parents’ variant profiles to know the family’s inherited pattern and discovered that his mother (I-2) also had p.(Trp3136Cys) in *MYO15A* while his father (I-1) had p.(Phe3420del) in *MYO15A* (Table 2). Notably, p.(Phe3420del) in *MYO15A* has been reported in hearing loss patients from the Chinese Han population [17].

**Table 2:** Variants identified in the parents of DE3386 (DE4451 and DE4452).

Case	Gene	Variant type	Inheritance	Nucleotide change	Protein change	Genotype	SIFT	Polyphen2	Mutation Taster	1000 Genomes ASN	ExAC ASN	Normal control (allele fre, %)	Pathogenicity (Deafness variation database^a^)	AMA and ACMG guidelines
**DE4451**	*MYO15A*	Missense	AR	NM_016239.3:c.9408G>C	NP_057323.3:p.(Trp3136Cys)	Het	D	D	D	0	0.0002	0	-	Likely pathogenic
	*MYO15A*	Missense	AR	NM_016239.3:c.4457G>T	NP_057323.3:p.(Gly1486Val)	Het	D	D	D	0	0	0	-	Likely pathogenic
**DE4452**	*MYO15A*	Deletion	AR	NM_016239.3:c.10258_10260del	NP_057323.3:p.(Phe3420del)	Het	.	.	.	0	0	0	Pathogenic	Likely pathogenic
	*MYO15A*	Missense	AR	NM_016239.3:c.4101C>A	NP_057323.3:p.(Asn1367Lys)	Het	D	D	D	0	0	0	-	Likely pathogenic
** **	*MYH14*	Missense	AD	NM_024729.3:c.2080C>T	NP_079005.3:p.(Arg694Cys)	Het	D	D	D	0	0	0	-	Likely pathogenic

^a^ Deafness variation database

B: Benign; T: Tolerated; D: Damaging/deleterious; PD: Probably damaging; NA: Not available; N: Polymorphism

VUS: Variant of uncertain significance

AD: Autosomal dominant; AR: Autosomal recessive

### Correlation of genotype and CI outcomes in patients with candidate variants

The CI outcomes of the 10 patients who harbored the identified candidate variants are listed in Table 3. All patients had received CI at least 4.9 years prior to enrolling in our study. Among them, patients DE3221 and DE4702 had variants in *COL11A2* and *TMC1*, respectively, and were classified as having poor outcomes. Conversely, patient DE4377, who also harbored a variant in *TMC1*, showed a good CI outcome. The remaining seven patients had variants in *MYO15A*, *MYO3A*, *MYH14*, *ACTG1*, *WFS1*, *DSPP*, and *GRHL2*, and tended to have good outcomes (CAP and SIR scores of more than 5 and 3, respectively).

**Table 3:** CI outcomes of the 10 patients with the identified candidate variants.

Sample ID	Sex	Genotype	Age at implantation, yr	Preoperative hearing, dBHL	Duration of rehabilitation, yr	CAP	SIR
DE2864	F	*WFS1* p.(Ala58Val)/ WT	5.6	100	6.4	5	4
DE3221	M	*COL11A2* p.(Arg64Gln)/ WT	1.7	120	6.5	4	2
DE3241	M	*MYO3A* p.(Ile419Thr)/ WT	25.1	N/A	12	5	4
DE3335	M	*DSPP* p.(Asp1008Asn) / WT	4	120	4.9	6	4
DE3386	F	*MYH14* p.(Arg694Cys)/ WT	1.5	106.6	5.7	5	4
		*MYO15A* p.(Gly1486Val)/ p.(Asn1367Lys)					
DE3395	F	*ACTG1* p.(Thr277Ile)/ WT	26.3	108.3	6	6	5
DE4372	M	*MYO15A* p.(Gln1815Lys)/ p.(Arg1993Trp)	2.3	116.6	12.1	6	5
DE4377	F	*TMC1* p.(Phe544Leu)/ WT	9.6	101.6	14.6	6	5
DE4467	F	*GRHL2* p.(Gly65Ser)/ WT	4.2	90	14	6	5
DE4702	M	*TMC1* p.(Phe593Leu)/ WT	5.5	100	13	4	3

CAP: Categories of Auditory Performance

SIR: Speech Intelligibility Rating

## Discussion

In the present study, we sought to clarify the genetic characteristics of non-syndromic deafness patients without the known mutations in common deafness-related genes. Using MPS, we successfully identified candidate variants in 24.4% of these patients (10/41) by targeting 40 relatively less common and non-syndromic hearing loss genes. We also examined the segregation of the potential disease-causing variants in the families of two multiplex probands. Among the patients harboring the identified candidate variants, eight showed good CI outcomes, while two showed poor outcomes.

Several studies conducted in Eastern Asia have detected deafness genes at different rates in various targeted cohorts. For example, Wu et al. found that approximately 20.6% (37/180) of Taiwanese children with CI exhibited mutations in four common deafness-related genes: *GJB2*, *SLC26A4*, the mitochondrial 12S rRNA gene, and *OTOF* [14]. A phenotype-driven candidate gene approach of screening 204 reported hearing loss-related genes followed by targeted resequencing was used to find causative variants in 54.8% of sporadic severe-to-profound hearing loss patients in a Korean population [28]. In another study, MPS was used to genetically screen 60% and 36% of patients with prelingual onset hearing loss and postlingual onset hearing loss, respectively, in a Japanese population [25]. Another study used a comprehensive deafness gene panel (including 50 non-syndromic and 7 non-syndromic/syndromic deafness genes) to screen 125 deaf probands without common mutations in *GJB2*, *SLC26A4*, or *MT-RNR1*, and found potentially causative mutations in 26.4% (33/125) of the patients [17]. Collectively, these studies showed that different targeted genes and objects were selected at different detection rates in Asian populations, and that genetic causes play important roles in different classes of deafness. However, two limitations should be noted with respect to our study: (1) we examined a relatively small population, and (2) our utilized genetic panel did not include all of the reported non-syndromic deafness genes. Thus, some relevant variations may exist in untargeted regions (e.g., introns or regulatory elements) of screened genes and/or untargeted genes. That said, our results could facilitate the design or updating of genetic screening panels that include both common and rare deafness-related variants. Future studies could seek to increase the sample size of non-syndromic hearing loss patients and/or seek to design a new sequencing panel for clinical genomic diagnosis.

Our study population included the probands of two deafness-segregating families in which additional family members agreed to participate, allowing us to examine the potential clinical significance of the candidate variants by performing linkage analysis [40]. *ACTG1* p.(Thr277Ile) was identified and confirmed in Family DE3395. *ACTG1* encodes γ-actin, which is the predominant actin isoform in auditory hair cells, particularly those of the cuticular plate, adherens junction, and stereocilia [41, 42], and mutations in this gene have been reported to cause the autosomal dominant sensorineural hearing loss, DFNA20/26 [41, 42]. Previous reports found that the onset age of hearing loss in most cases caused by *ACTG1* mutation is in the first decade or second decade of life [20, 43–46]. A similar pattern was seen in DE3395 (II-2) and his affected family members (I-2, II-1, and II-3). We found that three of the four affected family members exhibited the p.(Thr277Ile) variant, with the remaining affected individual (II-3) refusing to participate in genetic screening. The daughter of DE3395 (III-1) was found to have inherited the candidate variant; she currently has normal hearing, but her age (8 years at the time of screening) suggests that the phenotype may not have appeared yet. Thus, she has a high possibility of experiencing progressive hearing loss in the future. The family of III-1 should be counseled to have her hearing ability checked periodically. It is conceivable that patients with CIs could be expected to acquire a good outcome because *ACTG1* acts in the hair cells of the cochlea.

In the proband of the second multiplex family, DE3386, we identified three candidate variants, *MYH14* p.(Arg694Cys) (heterozygous variant), *MYO15A* p.(Gly1486Val) (compound heterozygous variant), and *MYO15A* p.(Asn1367Lys) (compound heterozygous variant). Mutations in *MYH14*, which encodes a long-chain non-muscle myosin IIC protein, were previously reported as causative for ADSNHI and DFNA4 [47–49]. *MYH14* gene expression is detected in the organ of Corti and the stria vascularis in the inner ear; and Hensen cells, Claudius cells, and external sulcus cells which are surrounding the cochlea, all of which are important for the neural system that enables hearing [48]. Although the p.(Arg694Cys) variant is located in a non-functional domain, our findings suggest that additional work may be warranted to examine its potential impact. *MYO15A* (DFNB3) has been reported as a causative gene for ARSNHI [50]; it encodes myosin XV, which is an actin-dependent molecular motor family member that can hydrolyze ATP to enable actin filament movement [51]. The amino acids altered by the p.(Gly1486Val) and p.(Asn1367Lys) are located in the myosin motor domain and might impact the protein function in patient DE3386. Since this individual harbored three potentially relevant variants, we were unable to determine whether his deafness phenotype was due to the compound heterozygous variants in *MYO15A* and/or the heterozygous variant in *MYH14*. As the parents of DE3389 both had severe hearing loss (Fig 3A), we recruited them for additional analysis. Direct Sanger sequencing provided limited results: each parent was heterozygous for one of the variants in *MYO15A*, while the father additionally carried the *MYH14* variant. Further MPS-based screening of the parents with our panel revealed that each had an additional variant in *MYO15A*: the father had p.(Phe3420del), which was reported as a pathogenic allele in a Chinese Han population [17], and the mother had p.(Trp3136Cys) in *MYO15A*, which is a novel candidate variant identified herein. This demonstrates that the MPS technique is a powerful tool for identifying genetic defects in hereditary hearing loss.

Several studies in CI patients have demonstrated that those with certain deafness gene mutations showed different treatment responses [14, 20, 24, 25]. For example, mutations in *PCDH15* or *DFNB59* tended to be associated with poor CI outcomes [24], whereas good outcomes were usually seen in patients with mutations in *MYO6*, *ACTG1*, and or *MYO15A* who received electric acoustic stimulation (EAS) and CI [21–23]. Here, we observed good CI performance in patients with variants in *MYO15A*, *MYO3A*, *MYH14*, *ACTG1*, *WFS1*, *DSPP*, and *GRHL2*. Some reports have described good performance in CI patients with mutations in *MYO15A* [20, 21], *MYO3A* [52], *ACTG1* [22], and *WFS1* [53]. However, our patient DE3221, who had a variant in *COL11A2* (Table 3), had a poor CI outcome. Among our patients, there was a discrepancy in the CI outcomes of two patients with variants in *TMC1*. Although this gene is expressed in inner and outer hair cells, it does not correspond to our current hypothesis about the relationship of CI outcomes and location of mutated genes. Moreover, as we did not examine the inheritance mode(s) of the *TMC1* variants in patients DE4377 and DE4702, it remains possible that one could represent a dominantly inherited pathogenic variant while the other was a recessively inherited carrier allele. We also note that environmental factors might influence the success of CI, leading to unexpected results. Nevertheless, most of the previous studies and our present results are consistent with the idea that the treatment outcome can be roughly predicted from the expression pattern of the variant protein, with a good outcome generally seen for variants in proteins expressed in portions on the cochlea, while a poor outcome seems more likely when the variant is in a protein related to sensory neurons. In the future, this general paradigm could help clinicians and patients make strategic decisions prior to beginning treatment.

In conclusion, we herein used MPS and a filtering strategy to identify candidate variants of rare deafness-related genes in SNHI patients. We also examined potential correlations between the identified variants and CI performance. Our findings support the idea that genetic examination could help predict the performance of implantation, which could assist deaf patients and clinicians in deciding whether or not pursue surgery.

## Supporting information

S1 Table The 40 rare SNHI-related genes examined in this study. (XLSX)

## References

[1] Morton CC, Nance WE. Newborn hearing screening—a silent revolution. The New England journal of medicine. 2006;354(20):2151–64. 10.1056/NEJMra050700 16707752

[2] Niparko JK, Tobey EA, Thal DJ, Eisenberg LS, Wang NY, Quittner AL, et al Spoken language development in children following cochlear implantation. JAMA. 2010;303(15):1498–506. 10.1001/jama.2010.451 20407059 PMC3073449

[3] Nikolopoulos TP, O'Donoghue GM, Archbold S. Age at implantation: its importance in pediatric cochlear implantation. Laryngoscope. 1999;109(4):595–9. 10.1097/00005537-199904000-00014 10201747

[4] Waltzman SB, Roland JT Jr. Cochlear implantation in children younger than 12 months. Pediatrics. 2005;116(4):e487–93. 10.1542/peds.2005-0282 16199675

[5] Cullen RD, Higgins C, Buss E, Clark M, Pillsbury HC 3rd, Buchman CA. Cochlear implantation in patients with substantial residual hearing. Laryngoscope. 2004;114(12):2218–23. 10.1097/01.mlg.0000149462.88327.7f 15564849

[6] Papsin BC. Cochlear implantation in children with anomalous cochleovestibular anatomy. Laryngoscope. 2005;115(1 Pt 2 Suppl 106):1–26. 10.1097/00005537-200501001-00001 15626926

[7] Walton J, Gibson WP, Sanli H, Prelog K. Predicting cochlear implant outcomes in children with auditory neuropathy. Otol Neurotol. 2008;29(3):302–9. 10.1097/MAO.0b013e318164d0f6 18317399

[8] Rennels M, Pickering LK. Sensorineural hearing loss in children. Lancet. 2005;365(9477):2085–6. 10.1016/S0140-6736(05)66724-4 15964436

[9] Hilgert N, Smith RJ, Van Camp G. Forty-six genes causing nonsyndromic hearing impairment: which ones should be analyzed in DNA diagnostics? Mutat Res. 2009;681(2–3):189–96. 10.1016/j.mrrev.2008.08.002 18804553 PMC2847850

[10] Wu CC, Chen PJ, Chiu YH, Lu YC, Wu MC, Hsu CJ. Prospective mutation screening of three common deafness genes in a large Taiwanese Cohort with idiopathic bilateral sensorineural hearing impairment reveals a difference in the results between families from hospitals and those from rehabilitation facilities. Audiol Neurootol. 2008;13(3):172–81. 10.1159/000112425 18075246

[11] Wu CC, Lee YC, Chen PJ, Hsu CJ. Predominance of genetic diagnosis and imaging results as predictors in determining the speech perception performance outcome after cochlear implantation in children. Arch Pediatr Adolesc Med. 2008;162(3):269–76. 10.1001/archpediatrics.2007.59 18316665

[12] Dahl HH, Wake M, Sarant J, Poulakis Z, Siemering K, Blamey P. Language and speech perception outcomes in hearing-impaired children with and without connexin 26 mutations. Audiol Neurootol. 2003;8(5):263–8. 10.1159/000071998 12904681

[13] Sinnathuray AR, Raut V, Awa A, Magee A, Toner JG. A review of cochlear implantation in mitochondrial sensorineural hearing loss. Otol Neurotol. 2003;24(3):418–26. 12806294 10.1097/00129492-200305000-00012

[14] Wu CC, Liu TC, Wang SH, Hsu CJ, Wu CM. Genetic characteristics in children with cochlear implants and the corresponding auditory performance. Laryngoscope. 2011;121(6):1287–93. 10.1002/lary.21751 21557232

[15] Nishio SY, Usami S. Deafness gene variations in a 1120 nonsyndromic hearing loss cohort: molecular epidemiology and deafness mutation spectrum of patients in Japan. Ann Otol Rhinol Laryngol. 2015;124 Suppl 1:49S–60S. 25788563 10.1177/0003489415575059

[16] Shearer AE, DeLuca AP, Hildebrand MS, Taylor KR, Gurrola J 2nd, Scherer S, et al Comprehensive genetic testing for hereditary hearing loss using massively parallel sequencing. Proc Natl Acad Sci U S A. 2010;107(49):21104–9. 10.1073/pnas.1012989107 21078986 PMC3000272

[17] Yang T, Wei X, Chai Y, Li L, Wu H. Genetic etiology study of the non-syndromic deafness in Chinese Hans by targeted next-generation sequencing. Orphanet J Rare Dis. 2013;8:85 10.1186/1750-1172-8-85 23767834 PMC3703291

[18] Miyagawa M, Naito T, Nishio SY, Kamatani N, Usami S. Targeted exon sequencing successfully discovers rare causative genes and clarifies the molecular epidemiology of Japanese deafness patients. PLoS One. 2013;8(8):e71381 10.1371/journal.pone.0071381 23967202 PMC3742761

[19] Wu CC, Lin YH, Lu YC, Chen PJ, Yang WS, Hsu CJ, et al Application of massively parallel sequencing to genetic diagnosis in multiplex families with idiopathic sensorineural hearing impairment. PLoS One. 2013;8(2):e57369 10.1371/journal.pone.0057369 23451214 PMC3579845

[20] Miyagawa M, Nishio SY, Ikeda T, Fukushima K, Usami S. Massively parallel DNA sequencing successfully identifies new causative mutations in deafness genes in patients with cochlear implantation and EAS. PLoS One. 2013;8(10):e75793 10.1371/journal.pone.0075793 24130743 PMC3794008

[21] Miyagawa M, Nishio SY, Hattori M, Moteki H, Kobayashi Y, Sato H, et al Mutations in the MYO15A gene are a significant cause of nonsyndromic hearing loss: massively parallel DNA sequencing-based analysis. Ann Otol Rhinol Laryngol. 2015;124 Suppl 1:158S–68S. 25792667 10.1177/0003489415575058

[22] Miyagawa M, Nishio SY, Ichinose A, Iwasaki S, Murata T, Kitajiri S, et al Mutational spectrum and clinical features of patients with ACTG1 mutations identified by massively parallel DNA sequencing. Ann Otol Rhinol Laryngol. 2015;124 Suppl 1:84S–93S. 25792668 10.1177/0003489415575057

[23] Miyagawa M, Nishio SY, Kumakawa K, Usami S. Massively parallel DNA sequencing successfully identified seven families with deafness-associated MYO6 mutations: the mutational spectrum and clinical characteristics. Ann Otol Rhinol Laryngol. 2015;124 Suppl 1:148S–57S. 25999546 10.1177/0003489415575055

[24] Wu CC, Lin YH, Liu TC, Lin KN, Yang WS, Hsu CJ, et al Identifying Children With Poor Cochlear Implantation Outcomes Using Massively Parallel Sequencing. Medicine (Baltimore). 2015;94(27):e1073. 26166082 10.1097/MD.0000000000001073 PMC4504554

[25] Miyagawa M, Nishio SY, Usami S. A Comprehensive Study on the Etiology of Patients Receiving Cochlear Implantation With Special Emphasis on Genetic Epidemiology. Otol Neurotol. 2016;37(2):e126–34. 10.1097/MAO.0000000000000936 26756145 PMC4710159

[26] Eppsteiner RW, Shearer AE, Hildebrand MS, Deluca AP, Ji H, Dunn CC, et al Prediction of cochlear implant performance by genetic mutation: the spiral ganglion hypothesis. Hear Res. 2012;292(1–2):51–8. 10.1016/j.heares.2012.08.007 22975204 PMC3461332

[27] Wu CC, Lu YC, Chen PJ, Liu AY, Hwu WL, Hsu CJ. Application of SNaPshot multiplex assays for simultaneous multigene mutation screening in patients with idiopathic sensorineural hearing impairment. Laryngoscope. 2009;119(12):2411–6. 10.1002/lary.20621 19718752

[28] Chang MY, Choi BY. Strategy for the customized mass screening of genetic sensorineural hearing loss in koreans. Korean J Audiol. 2014;18(2):45–9. 10.7874/kja.2014.18.2.45 25279224 PMC4181059

[29] Chang X, Wang K. wANNOVAR: annotating genetic variants for personal genomes via the web. J Med Genet. 2012;49(7):433–6. 10.1136/jmedgenet-2012-100918 22717648 PMC3556337

[30] Yang H, Wang K. Genomic variant annotation and prioritization with ANNOVAR and wANNOVAR. Nat Protoc. 2015;10(10):1556–66. 10.1038/nprot.2015.105 26379229 PMC4718734

[31] Genomes Project C, Abecasis GR, Auton A, Brooks LD, DePristo MA, Durbin RM, et al An integrated map of genetic variation from 1,092 human genomes. Nature. 2012;491(7422):56–65. 10.1038/nature11632 23128226 PMC3498066

[32] Lek M, Karczewski KJ, Minikel EV, Samocha KE, Banks E, Fennell T, et al Analysis of protein-coding genetic variation in 60,706 humans. Nature. 2016;536(7616):285–91. 10.1038/nature19057 27535533 PMC5018207

[33] Ng PC, Henikoff S. SIFT: Predicting amino acid changes that affect protein function. Nucleic acids research. 2003;31(13):3812–4. 12824425 10.1093/nar/gkg509 PMC168916

[34] Adzhubei IA, Schmidt S, Peshkin L, Ramensky VE, Gerasimova A, Bork P, et al A method and server for predicting damaging missense mutations. Nature methods. 2010;7(4):248–9. 10.1038/nmeth0410-248 20354512 PMC2855889

[35] Schwarz JM, Rodelsperger C, Schuelke M, Seelow D. MutationTaster evaluates disease-causing potential of sequence alterations. Nature methods. 2010;7(8):575–6. 10.1038/nmeth0810-575 20676075

[36] Archbold S, Lutman ME, Nikolopoulos T. Categories of auditory performance: inter-user reliability. Br J Audiol. 1998;32(1):7–12. 9643302 10.3109/03005364000000045

[37] Allen C, Nikolopoulos TP, Dyar D, O'Donoghue GM. Reliability of a rating scale for measuring speech intelligibility after pediatric cochlear implantation. Otol Neurotol. 2001;22(5):631–3. 11568670 10.1097/00129492-200109000-00012

[38] Kurima K, Peters LM, Yang Y, Riazuddin S, Ahmed ZM, Naz S, et al Dominant and recessive deafness caused by mutations of a novel gene, TMC1, required for cochlear hair-cell function. Nat Genet. 2002;30(3):277–84. 10.1038/ng842 11850618

[39] Richards S, Aziz N, Bale S, Bick D, Das S, Gastier-Foster J, et al Standards and guidelines for the interpretation of sequence variants: a joint consensus recommendation of the American College of Medical Genetics and Genomics and the Association for Molecular Pathology. Genet Med. 2015;17(5):405–24. 10.1038/gim.2015.30 25741868 PMC4544753

[40] Brownstein Z, Bhonker Y, Avraham KB. High-throughput sequencing to decipher the genetic heterogeneity of deafness. Genome Biol. 2012;13(5):245 10.1186/gb-2012-13-5-245 22647651 PMC3446284

[41] Morin M, Bryan KE, Mayo-Merino F, Goodyear R, Mencia A, Modamio-Hoybjor S, et al In vivo and in vitro effects of two novel gamma-actin (ACTG1) mutations that cause DFNA20/26 hearing impairment. Hum Mol Genet. 2009;18(16):3075–89. 10.1093/hmg/ddp249 19477959 PMC2714729

[42] Khaitlina SY. Functional specificity of actin isoforms. Int Rev Cytol. 2001;202:35–98. 11061563 10.1016/s0074-7696(01)02003-4

[43] Rendtorff ND, Zhu M, Fagerheim T, Antal TL, Jones M, Teslovich TM, et al A novel missense mutation in ACTG1 causes dominant deafness in a Norwegian DFNA20/26 family, but ACTG1 mutations are not frequent among families with hereditary hearing impairment. Eur J Hum Genet. 2006;14(10):1097–105. 10.1038/sj.ejhg.5201670 16773128

[44] Liu P, Li H, Ren X, Mao H, Zhu Q, Zhu Z, et al Novel ACTG1 mutation causing autosomal dominant non-syndromic hearing impairment in a Chinese family. J Genet Genomics. 2008;35(9):553–8. 10.1016/S1673-8527(08)60075-2 18804074

[45] de Heer AM, Huygen PL, Collin RW, Oostrik J, Kremer H, Cremers CW. Audiometric and vestibular features in a second Dutch DFNA20/26 family with a novel mutation in ACTG1. Ann Otol Rhinol Laryngol. 2009;118(5):382–90. 10.1177/000348940911800511 19548389

[46] van Wijk E, Krieger E, Kemperman MH, De Leenheer EM, Huygen PL, Cremers CW, et al A mutation in the gamma actin 1 (ACTG1) gene causes autosomal dominant hearing loss (DFNA20/26). J Med Genet. 2003;40(12):879–84. 10.1136/jmg.40.12.879 14684684 PMC1735337

[47] Lalwani AK, Goldstein JA, Kelley MJ, Luxford W, Castelein CM, Mhatre AN. Human nonsyndromic hereditary deafness DFNA17 is due to a mutation in nonmuscle myosin MYH9. American journal of human genetics. 2000;67(5):1121–8. 10.1016/S0002-9297(07)62942-5 11023810 PMC1288554

[48] Donaudy F, Snoeckx R, Pfister M, Zenner HP, Blin N, Di Stazio M, et al Nonmuscle myosin heavy-chain gene MYH14 is expressed in cochlea and mutated in patients affected by autosomal dominant hearing impairment (DFNA4). Am J Hum Genet. 2004;74(4):770–6. 10.1086/383285 15015131 PMC1181955

[49] Mhatre AN, Li J, Kim Y, Coling DE, Lalwani AK. Cloning and developmental expression of nonmuscle myosin IIA (Myh9) in the mammalian inner ear. Journal of neuroscience research. 2004;76(3):296–305. 10.1002/jnr.20065 15079858

[50] Wang A, Liang Y, Fridell RA, Probst FJ, Wilcox ER, Touchman JW, et al Association of unconventional myosin MYO15 mutations with human nonsyndromic deafness DFNB3. Science. 1998;280(5368):1447–51. 9603736 10.1126/science.280.5368.1447

[51] Krendel M, Mooseker MS. Myosins: tails (and heads) of functional diversity. Physiology. 2005;20:239–51. 10.1152/physiol.00014.2005 16024512

[52] Walsh T, Walsh V, Vreugde S, Hertzano R, Shahin H, Haika S, et al From flies' eyes to our ears: mutations in a human class III myosin cause progressive nonsyndromic hearing loss DFNB30. Proc Natl Acad Sci U S A. 2002;99(11):7518–23. 10.1073/pnas.102091699 12032315 PMC124268

[53] Hogewind BF, Pennings RJ, Hol FA, Kunst HP, Hoefsloot EH, Cruysberg JR, et al Autosomal dominant optic neuropathy and sensorineual hearing loss associated with a novel mutation of WFS1. Mol Vis. 2010;16:26–35. 20069065 PMC2805421

